# Diagnosis, Symptoms, and Outcomes of Hirschsprung's Disease from the Perspective of Gender

**DOI:** 10.1155/2017/9274940

**Published:** 2017-03-07

**Authors:** Christina Granéli, Eero Dahlin, Anna Börjesson, Einar Arnbjörnsson, Pernilla Stenström

**Affiliations:** Department of Pediatric Surgery, Skåne University Hospital and Institution of Clinical Research, Lund University, Lund, Sweden

## Abstract

*Background/Aim*. Hirschsprung's disease (HD) has a skewed gender distribution, with a female to male ratio of 1 : 4. This study aims to examine differences between boys and girls with HD regarding preoperative features and postoperative treatment and outcome.* Method*. The first part of the study was conducted as a retrospective review of all HD patients who underwent transanal endorectal pull-through (TERPT). Pre-, peri-, immediate post-, and first-year postoperative data were collected. The second part was conducted as an observational cross-sectional study by comparing bowel function scores (BFS) determined by structured interviews of patients 4 years old and older.* Results*. Included were 39 boys and 12 girls. Of these, 25 boys and 9 girls were older than 4 years and participated in the BFS interview. Boys had a higher frequency of hospitalizations during the first postoperative year compared to girls (*n* = 20 and *n* = 2, *p* < 0.05). At long-term follow-up, more boys reported abnormal frequency of defecation, 16 compared to 2 (*p* < 0.05). There was no difference between genders in terms of preoperative symptoms and overall bowel function later.* Conclusion*. Boys with HD had more hospitalizations and a higher rate of abnormal frequency of defecation than girls with HD.

## 1. Introduction

Hirschsprung's disease (HD) is a congenital disorder with a reported prevalence of 1 : 5000 and a female to male ratio of 1 : 4 [1, 2]. Because it is a rare disease, studies often include only small numbers of patients, without gender-specific analyses. Patients with HD are usually diagnosed and undergo surgery during the neonatal period. Transanal endorectal pull-through (TERPT) has become one of the most common surgical procedures for HD in recent decades [3]. Both short-term outcomes after TERPT, such as anastomotic leakage, bowel obstruction, and perineal excoriation, and long-term outcomes regarding bowel function have been evaluated in gender-mixed groups [4–8].

In recent years gender itself has come into greater focus as an important factor also in children with other diseases than HD. Pre-, peri-, and postoperative results after pediatric surgeries and outcomes after heart surgery in children have been analyzed from the perspective of gender [9, 10]. Similar gender-specific analyses of children with HD could contribute to a greater understanding of patient characteristics in terms of gender-typical outcomes and postoperative needs.

The primary aim with this study was to determine whether there were differences between boys and girls with HD in terms of preoperative features, operative course, and first-year postoperative results. The secondary aim was to assess patients for gender-specific differences in bowel control after 4 years of age.

## 2. Materials and Methods

### 2.1. Setting

The study was performed at a tertiary center for pediatric surgery serving a region with 2 million residents and 23,000 births per year. The study was retrospective regarding preoperative information and short-term complications, and cross-sectional regarding bowel function as a long-term outcome.

### 2.2. Patients and Diagnoses

**Figure 1:**
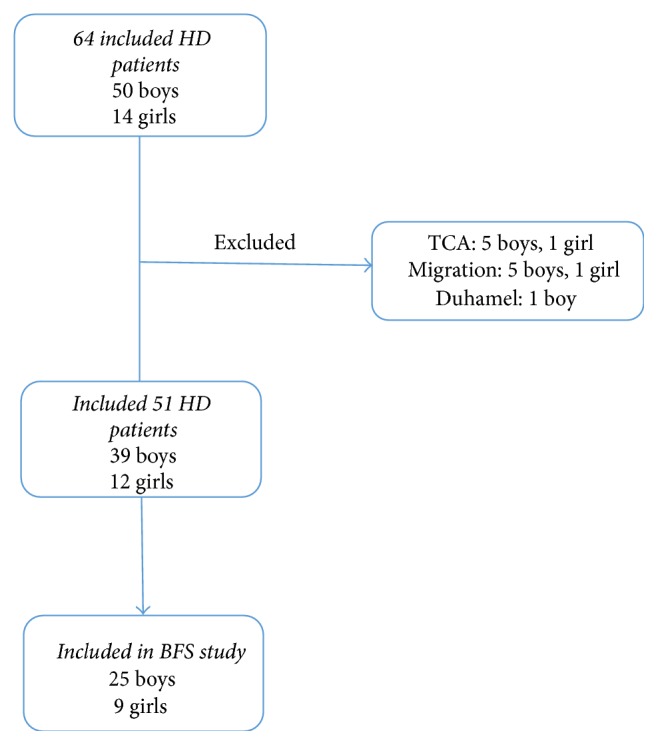
Flowchart of patients with Hirschsprung's disease (HD) and exclusions. Total colonic aganglionosis (TCA).

Patients who were diagnosed with HD and underwent TERPT from July 2005 through December 2015 were included. Patients who had migrated to or from the region after TERPT and those with total colonic aganglionosis (TCA) were excluded (Figure 1).

Anography with cold contrast provided information about absence of the rectoanoinhibition reflex as well as the length of affected bowel [11], and this in combination with a rectal biopsy confirmed the diagnosis of HD in all included patients.

### 2.3. Peri- and Postoperative Data

The patients' medical records were retrospectively reviewed and data including birth weight, gestational week, presenting symptoms, and diagnosis were compiled. Surgical data and immediate postoperative results were also reviewed. The number of scheduled and emergency outpatient visits related to the HD diagnosis during the first year after TERPT and the total number of calibrations, dilations, and additional operations up to the time for long-term follow-up were recorded. The number of treatments with onabotulinumtoxinA injected into the anal sphincter when outlet symptoms were present was also noted.

### 2.4. Scoring of Bowel Symptoms

The HD patients who were 4 years old and older and their guardians were interviewed and bowel function was assessed according to a structured bowel function score (BFS) in which bowel function from worse to better is scored on a scale from 1 to 20 (1 being very poor and 20 being very good) [5]. A BFS of >17 has previously been defined as good/normal bowel function [5, 12]. The interviews were performed during regular counseling and follow-up either at the outpatient clinic or by telephone.

### 2.5. Surgical Technique

The TERPT procedure was performed in accordance with the technique described in 1998, with rectal mucosectomy, colectomy of the aganglionic segment, and anal pull-through of the normoganglionic colon [3]. The length of the muscular cuff was 2 to 3 centimeters. Colon resection was extended to include the transition zone, and any dilated bowel was resected along with the aganglionic bowel. Frozen-section biopsy of the end of the proximal bowel was performed during surgery to confirm the presence of a normal frequency of mature ganglionic cells without signs of nerve hypertrophy. The final pathology report included results of calretinin staining. In cases where the extent of aganglionosis was unclear on anography or when the length of affected bowel was >20 cm, a laparoscopic approach was used to free the left flexure and/or to permit immediate frozen-section biopsies. A preoperative stoma had been established in cases where the initial diagnosis of HD was unclear or when patients had enterocolitis that was resistant to medical treatment and colonic washout. All TERPT procedures were performed by 3 pediatric colorectal surgeons.

### 2.6. Definitions

Delayed passage of meconium was defined as that occurring later than 48 h after birth [13].

Enterocolitis was defined according to intention to treat in the presence of distended bowel, fever, and foul-smelling stool along with positivity for serum inflammatory markers [14].

Dilation was defined as a widening in the anastomotic area under general anesthesia. Calibration was defined according to Hegar size of the anal canal assessed in the outpatient clinic.

### 2.7. Statistical Analysis

The study design was formulated by a statistician. We planned a study of independent cases, boys, and controls, girls, with 0.3 control(s) per case. The intention was to disclose large differences, 50%, between the measured parameters. Prior data indicate that the probability of exposure among controls is 0.3. If the true probability of exposure among cases is 0.8, we will need to study 37 case patients and 11 control patients to be able to reject the null hypothesis that the exposure rates for case and controls are equal with probability (power) 0.8. The Type I error probability associated with this test of this null hypothesis is 0.05. We used the Fisher's exact test to evaluate this null hypothesis.

Fisher's two-tailed exact test was used for categorical outcomes and the Mann–Whitney *U* test for numerical outcomes. *p* values < 0.05 were considered significant. Calculations were performed using Microsoft Excel and outcomes for numerical variables were presented as median (range).

### 2.8. Ethics

The protocol was designed to meet the legislative documentation required in the country of origin, and the regional research committee approved the study (registration number 2010/49). Informed consent was obtained from guardians of all patients who were included in the study. Intention to treat was the main diagnostic strategy used for all patients. All evaluations, treatments, and procedures described in this report met the established standards of care.

## 3. Results

### 3.1. Patient Characteristics

A total of 64 children were diagnosed with HD and/or treated for HD during the study period. Of these 50 (78%) were boys and 14 (22%) were girls. Excluded were patients with TCA, who migrated and one who was operated on with Duhamel (Figure 1). Thus, a total of 51 patients (39 boys and 12 girls) were included in the chart review portion of the study.

There were no significant differences between genders in birth weight or gestational week. Moreover, there were no differences in frequency of concomitant anomalies between girls and boys (Table 1).

### 3.2. Diagnosis

Diverting colostomy was performed before TERPT in 5 (12%, CI 4–26%) boys and 2 (17%, CI 2–48%) girls (*p* = 0.66). Boys had a colostomy for a median of 25 (range 2 to 377) days prior to TERPT and girls for a median of 6 (range 2 to 10) days before surgery (*p* = 0.86). There were no significant differences between genders in preoperative characteristics including age at symptom onset, duration of symptoms, age at biopsy, and final pathological diagnosis, or age at TERPT (Table 2). The most common initial symptoms in both boys and girls were delayed passage of meconium and bilious vomiting, without any gender difference (Table 3).

### 3.3. First-Year Postoperative Results

There was no significant difference between the length of the resected bowel between the boys 18 (10–114) and the girls 16 (13–27) cm (*p* = 0.76) (Table 4).

The length of postoperative hospital stay did not differ significantly between boys who stayed 4 days (range 1–22) and girls who stayed 3 days (range 2–7) (*p* = 0.067). The number of outpatient visits, either scheduled visits or emergency visits, also did not differ significantly between genders (Table 4).

Significantly higher percentage of boys were hospitalized during the first year compared with the girls (52% versus 17%, OR = 5.7, CI 1.004–61, *p* = 0.042). The frequency of hospitalizations in the whole cohort was significantly higher for the boys in comparison to the girls (1 versus 0, *p* < 0.03)

Anorectal complications including pain, skin irritation, excoriation, wounds, and fissures occurred in 16 (67%) boys and 5 (41%) girls (*p* = 0.17). There were no significant differences in frequency of calibration, dilation, or need for examination under anesthesia. Three boys (7%) and 1 (8%) girl were diagnosed with enterocolitis during the first year after TERPT.

There was one patient, a boy, who had a complication during the immediate postoperative course, which was leakage from a proximal biopsy site requiring a stoma.

### 3.4. Long-Term Follow-Up

The median duration of follow-up after TERPT was 4 (range 0.1–10) years for boys and 5 (range 0.5–9) years for girls in the whole cohort.

Appendicostomy was established in 4 (10%) boys and 3 (25%) girls (*p* = 0.3). In total, 3 boys and 0 girls required colostomy during the follow-up period (*p* = 1). Reasons for colostomy were urinary fistula that occurred in two boys (*n* = 2) and severe stricture in a boy with Down syndrome (*n* = 1). Two of these patients (1 fistula patient and 1 with stricture) had colostomy at the time of follow-up and were excluded from the BFS portion of the study. There were 2 (5%) boys and 1 (8%) girl who received treatment with onabotulinumtoxinA. Results are presented in Table 5.

### 3.5. Bowel Function

A total of 25 boys and 9 girls had reached the age of 4 years or older with intestinal continuity and were included in the BFS evaluation. The median duration of follow-up after TERPT was 7.0 (range 4.3 to 10.3) years for boys and 8.1 (range 4.3 to 9.9) years for girls. Three boys had Downs's syndrome and one boy had Bresheck syndrome and those four were excluded from the question about the social impact but included in the other questions regarding BFS as their parents could answer the questions. One girl had a translocation syndrome with cognitive dysfunction and was also excluded from the portion of the BFS regarding social impact.

The daily frequency of bowel movements differed significantly between boys, 15 (3–70) per week, and girls, 8 (5–21) per week (*p* < 0.05). Constipation was reported by 9 (36%) boys and 4 (44%) girls, and soiling with social impact (score 1 or lower) was present in 14 (56%) boys and 5 (55%) girls (Table 6).

The overall BFS was 14 (range 8 to 17) for boys and 13.5 (range 10 to 20) for girls (*p* = 0.34). Eighteen (86%) boys and 5 (56%) girls had BFS < 17 (*p* = 0.15).

## 4. Discussion

In this study on possible gender differences in patients with HD, it was found that more boys had hospitalizations during the first post-TERPT year, as compared to girls, despite the fact that there were no differences in other postoperative results between genders. In the long-term follow-up more boys than girls had abnormal frequency of defecation. There were no gender differences in terms of preoperative symptoms, treatment, or overall bowel function later on.

Boys had a somewhat longer postoperative hospital stay after TERPT than girls although not significantly. No previous study has presented data for in-hospital days for children with HD. However, in-hospital days for children with cardiovascular diseases have been analyzed. Contrary to our results, girls undergoing repair of congenital cardiac malformations had 18% more in-hospital days and greater 30-day postdischarge mortality rates than boys [9]. Klitzner et al. could not verify why exactly this was but speculated that the increased prevalence of comorbid medical conditions in females may indicate a larger role of biological factors rather than healthcare system variables or practice patterns was the answer. Another study within the Healthcare Cost and Utilization Project identified male gender as an independent predictor of increased resource utilization in patients with acute sinusitis [15]. Furthermore, in a study of preterm infants, male gender was associated with higher mortality and poorer long-term neurological outcomes prior to 27 weeks' gestational age [16], and a large retrospective study investigating both race and gender as factors in pediatric surgical outcomes that also implicated gender as an independent predictor of postoperative morbidity, length of stay, and total hospital charges showed that female gender seemed to have a favorable effect on postoperative morbidity [10]. That study had a cohort consisting of patients with various diagnoses, but it supports the idea that we should have gender in mind when presenting data regarding pediatric populations.

Several studies have evaluated clinical features and outcomes in patients with HD who have undergone TERPT, but the results were not analyzed separately for boys and girls. These studies have reported constipation rates that vary between 9 and 42% and soiling in 1 to 60% [6, 17–19]. The results for our patients were in the higher end of these spectra, with 36% of boys and 44% of girls reporting problems with constipation and 56% of boys and 55% of girls reporting soiling. The overall BFS among HD patients has been reported to be between 16 and 19 [5, 20], which is a better outcome than in our study. This could be because the median ages of patients in previous reports were older (15 and 43 years) [5, 20], while the median ages of patients in our study were 7 years (boys) and 8 years (girls). Also, children in our study were assessed by an independent researcher, and not by mail or by the operating physicians.

Enterocolitis was diagnosed in 7% of the boys and 8% of the girls during the first year after TERPT in our study, which was in the lower end of the spectrum compared to prior studies reporting rates of enterocolitis of 0% to 56% [6, 18, 21, 22].

The frequency of patients with appendicostomy was relatively high (10% among the boys and 25% among the girls) compared to previous literature. This could be due to the routine at our center, to provide patients with appendicostomy whenever their bowel management is unsatisfactory due to low compliance to a rectal approach for enemas. We also provide patients with appendicostomy already in preschool age in order to increase the children's autonomy early and to secure faecal continence before school start.

The main limitation of this study is the small number of patients. On the other hand, the strength is that there was a low drop-out rate. Residents of the study area are provided with free health care when needed, and therefore any strong attrition due to socioeconomic effects is unlikely. All of the patients who were included were closely followed and were treated by the same group of health care professionals, and the same structured forms were used to record the findings of regular visits, so that little information was lost in follow-up. The inclusion of children with syndromes and cognitive dysfunction could of course worsen the outcome but since the study intended to report on gender differences, and there was no difference in frequency of syndromes between boys and girls, we considered it to be most correct to include all patients with regard to their physical symptoms.

There are several confounding factors in the patients and the operative details studied. Furthermore, the number of patients in relation to the power of the statistics is rather low to determine whether the gender is really a significant factor. Thus, the significance of gender can be statistically determined in a small number of patients in case of a large difference only.

As to why outcomes in children with HD have not so far been evaluated in terms of gender, one might speculate that the less powerful statistics inherent to gender analysis in cohorts that are already limited in numbers could be a factor. It is also possible that researchers do not believe that there are gender differences among patients, socially or medically, until after puberty. However, gender differences may already be present during childhood due to anatomical differences, for example, in the pelvis and pelvic floor, or due to comorbidity. Different attitudes and different treatment according to gender have been demonstrated among adult patients [23], and until illuminated one cannot exclude that a similar difference in attitudes towards boys and girls exists among some health care professionals, and that this might influence treatment decisions and treatment outcomes. This possibility should warrant further study, not only among adults, but also among children.

## 5. Conclusion

Boys with HD have a higher rate of hospitalizations the first year after TERPT than girls with HD, and boys also have a higher frequency of bowel movements. Although analyzing patients according to gender might make it difficult to reach detectable statistical significance of results, we believe that outcomes for patients with HD should always be presented with regard to gender because awareness of possible gender differences might lead to better medical care and follow-up for both boys and girls.

## Figures and Tables

**Table 1:** Demographics of the included patients with HD who later underwent TERPT (%).

	Boys (*n*)		Girls (*n*)		*p*value^a^
*Birth week* median (range), weeks	39	39 (34–41)	12	40 (32–42)	0.12

*Birth weight* median (range), grams	38	3468 (2100–4675)	11	3490 (2800–4290)	0.24

Associated syndromes	8 (20)	7 Down's syndrome 1 Bresheck syndrome	2	1 translocation syndrome 1 combined syndrome	

Associated malformations	10	8 cardiac 1 ear 1 brain asymmetry 1 testicular 1 vertebrae	3	2 cardiac 1 skin	

^a^Mann–Whitney *U* test.

**Table 2:** Preoperative data presented as median (range) in days. The number of included patients was 39 boys and 12 girls; however information was missing in some of the patients' journals. PAD refers to Pathological Anatomical Diagnosis.

	Boys (*n* = 39)	Girls (*n* = 12)	*p*value^a^
Age at first symptom	1 (1–228)	1 (1–102)	0.22
Age at contact with pediatric surgeon	3 (1–1119)	3 (1–1054)	0.65
Duration of symptom	2 (0–952)	1 (0–952)	0.72
Age at biopsy	8 (1–1138)	10 (2–1054)	0.56
Age at histological diagnosis (PAD)	23 (7–1159)	23 (10–1072) *n* = 11	0.41
Time from histological diagnosis (PAD) to TERPT decision	5 (0–367)	3 (0–76) *n* = 11	0.54
Age at TERPT	47 (12–1279)	51 (15–1254)	0.46
Time from first contact with a pediatric surgeon to TERPT	37 (9–888)	43 (12–215)	0.39
Time from PAD to TERPT	19 (0–418)	30 (44–182) *n* = 11	0.30

^a^Mann–Whitney *U* test.

**Table 3:** Presenting symptoms.

	Boys (*n* = 39)	Girls (*n* = 12)	*p*value^a^
Delayed passage of meconium	29 (71%)	6 (50%)	0.30
Vomiting	24 (59%)	6 (50%)	0.74
Distended abdomen	18 (44%)	5 (42%)	1
Chronic constipation	8 (20%)	1 (8%)	0.67
Feeding problems	8 (20%)	4 (33%)	0.43
Fatigue	2 (5%)	2 (17%)	0.22
Radiology	2 (5%)	0	1
Weight loss	1 (2%)	0	1
Enterocolitis	2 (5%)	2 (17%)	0.22
Melena	0	1 (8%)	0.23

^a^Fisher exact test significant at *p* < 0.05.

**Table 4:** First-year postoperative and operative data, median (range), *n*: number. A total of 13 boys and 5 girls were admitted for emergency visits and a total of 20 boys and 2 girls were readmitted during the first year post-TERPT. The median value and the range are presented for the whole cohort, that is, both those who had emergency visits/or who were readmitted and those who were not.

	Boys (*n* = 39)	Girls (*n* = 12)	*p* value
Length of resected bowel (cm)	**18 (10–114)** *n* = 37	**16 (13–27)** *n* = 11	0.76^a^
Total number of follow-ups	8 (1–38)	7 (4–19)	0.73^a^
Planned visits	7 (1–21)	7 (4–14)	0.59^a^
Emergency visits	0 (0–11) *n* = 13	0 (0–5) *n* = 5	0.82^a^
Hospitalization	1 (0–6) *n* = 20	0 (0–4) *n* = 2	0.03^a^
Calibration	6 (1–26)	6 (1–13)	0.53^a^
Examinations in aesthesia	9 (23%)	2 (17%)	1^b^
Dilatation	0 (0–9)	0 (0–4)	0.46^a^
Anorectal complication	24 (67%)	5 (41%)	0.17^b^
Anal skin irritation/excoriation/wounds	16 (45%)	5 (41%)	1^b^

^a^Mann–Whitney  *U* test significant at *p* < 0.05.

^b^Fisher exact test significant at *p* < 0.05.

**Table 5:** Long-term follow-up data, median (range), *n*: number.

	Boys (*n* = 39)	Girls (*n* = 12)	*p* value
Follow-up (years)	4 (0.1–10)	5 (0.5–9)	0.1^a^
Complications	Urinary fistula *n* = 2 Stricture *n* = 1	0	1^b^
Appendicostomy	*n* = 4 (10%)	*n* = 3 (25%)	0.3^b^
Colostomy	*n* = 3 (8%)	*n* = 0 (0%)	1^b^
OnabotulinumtoxinA	*n* = 2 (5%)	*n* = 1 (8%)	0.56^b^

^a^Mann–Whitney  *U* test significant at *p* < 0.05.

^b^Fisher exact test significant at *p* < 0.05.

**Table 6:** Bowel Function Score (BFS). Patients 4 years of age and older. Ranging bowel function from worse to better with a score from 1 to 20 (1: very poor and 20: very good) (%).

Evaluation of bowel control	Score Min: 1 Max: 20	Boys *n* = 25	Girls *n* = 9	*p*value^a^
Ability to hold back defecation				
(i) Always	**3**	4 (16)	4 (44)	**0.21**
(ii) Problems < 1/week	**2**	12 (48)	2 (22)	
(iii) Weekly problems	**1**	7 (28)	2 (22)	
(iv) No voluntary control	**0**	2 (8)	1 (11)	

Feels/reports the urge to defecate				
(i) Always	**3**	1 (4)	4 (44)	**0.24**
(ii) Most of the time	**2**	11 (44)	0 (0)	
(iii) Uncertain	**1**	11 (44)	4 (44)	
(iv) Absent	**0**	2 (89)	1 (11)	

Frequency of defecation				
(i) Every other day to twice a day	**2**	9 (36)	7 (78)	**0.03**
(ii) More often	**1**	15 (60)	2 (22)	
(iii) Less often	**1**	1 (4)	0 (0)	

Soiling				
(i) Never	**3**	0 (0)	1 (11)	**0.34**
(ii) Staining < 1/week, no change of underwear required	**2**	11 (44)	3 (33)	
(iii) Frequent staining, change of underwear often required	**1**	9 (36)	4 (44)	
(iv) Daily soiling, requires protective aids	**0**	5 (20)	1 (11)	

Faecal accidents				
(i) Never	**3**	9 (36)	6 (67)	**0.15**
(ii) Fewer 1/week	**2**	9 (36)	1 (11)	
(iii) Weekly, requires protective aids	**1**	5 (20)	1 (11)	
(iv) Daily, requires protective aids day and night	**0**	2 (8)	1 (119)	

Constipation				
(i) No constipation	**3**	16 (64)	5 (56)	**0.24**
(ii) Manageable with diet	**2**	5 (20)	1 (11)	
(iii) Manageable with laxatives	**1**	4 (16)	1 (11)	
(iv) Manageable with enemas	**0**	0 (0)	2 (22)	

Social problems		*n* = 21	*n* = 8	
(i) No social problems	**3**	13 (62)	5 (63)	**0.5**
(ii) Sometimes	**2**	6 (29)	2 (25)	
(iii) Problems restricting social life	**1**	0 (0)	1 (12)	
(iv) Severe social/psychosocial problems	**0**	2 (9)	0 (0)	

Score		*n* = 21	*n* = 8	
Min Max Median		8 17 14	10 20 13.5	**0.36**

^a^Mann–Whitney  *U* test significant at *p* < 0.05.
